# Supplementary figures and images for: Molecular phylogeny of porcelain crabs (Porcellanidae: Petrolisthes and allies) from the south eastern Pacific: the genera Allopetrolisthes and Liopetrolisthes are not natural entities
Source: PeerJ. 2016 Mar 10;4:e1805. doi: 10.7717/peerj.1805 (PMC4793318; doi:10.7717/peerj.1805)

## A. Habit (dorsal view)

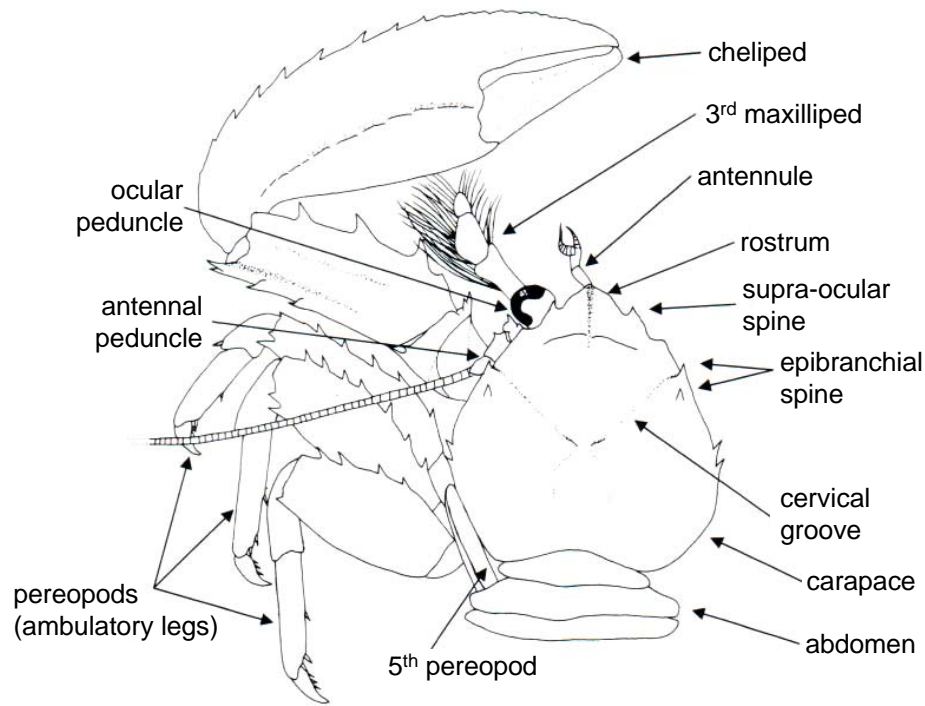

## B. Pereopod

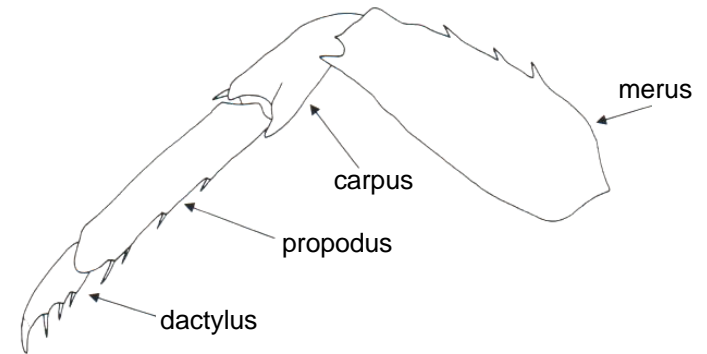

## D. Cheliped

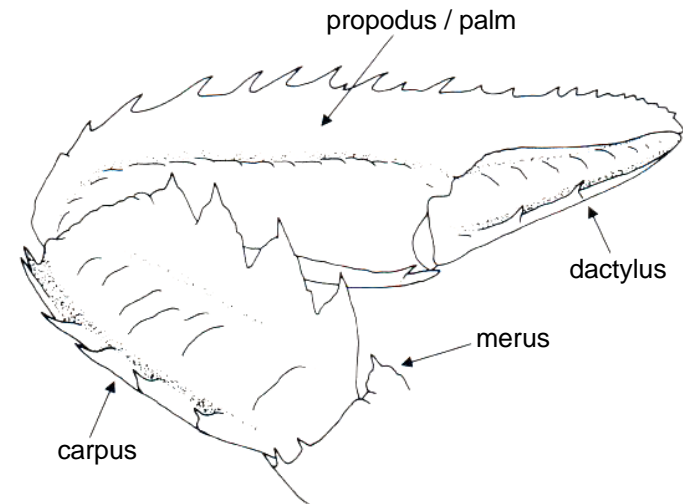

## C. Antenna

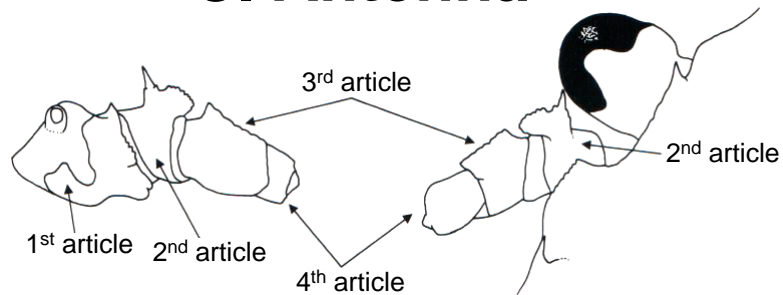

Supplement: Figure S1 — A. Crab habitus in dorsal view. B. Ambulatory leg (pereopod). C. Antenna. D. Cheliped. Modified from Osawa & Chan (2010). [file peerj-04-1805-s001.pdf]
